# A scoping review of self-supervised representation learning for clinical decision making using EHR categorical data

**DOI:** 10.1038/s41746-025-01692-1

**Published:** 2025-06-14

**Authors:** Yuanyuan Zheng, Adel Bensahla, Mina Bjelogrlic, Jamil Zaghir, Hugues Turbe, Lydie Bednarczyk, Christophe Gaudet-Blavignac, Julien Ehrsam, Stéphane Marchand-Maillet, Christian Lovis

**Affiliations:** 1 Division of Medical Information Sciences, Geneva University Hospitals, Geneva, Switzerland; 2 Department of Radiology and Medical Informatics, University of Geneva, Geneva, Switzerland; 3 Department of Computer Science, University of Geneva, Geneva, Switzerland

**Keywords:** Computer science, Machine learning, Predictive medicine

## Abstract

The widespread adoption of Electronic Health Records (EHRs) and deep learning, particularly through Self-Supervised Representation Learning (SSRL) for categorical data, has transformed clinical decision-making. This scoping review, following PRISMA-ScR guidelines, examines 46 studies published from January 2019 to April 2024, sourced from PubMed, MEDLINE, Embase, ACM, and Web of Science, focusing on SSRL for unlabeled categorical EHR data. The review systematically assesses research trends in building computationally and data-efficient representations for medical tasks, identifying major trends in model families: Transformer-based (43%), Autoencoder-based (28%), and Graph Neural Network-based (17%) models. The analysis highlights scenarios where healthcare institutions can leverage or develop SSRL technologies. It also addresses current limitations in assessing the impact of these technologies and identifies research opportunities to enhance their influence on clinical practice.

## Introduction

The advent of EHRs has revolutionized the healthcare industry by providing comprehensive, digitized patient information^1^. This shift has enabled healthcare providers to maintain accurate and accessible records, facilitating better patient care^2^. The widespread adoption of EHRs has fueled the development of deep learning models for various automated clinical decision-making, offering sophisticated tools for predicting patient trajectories, identifying disease patterns, and personalizing treatments^3,4^.

Recently, an increasing number of deep neural networks (DNNs) based on SSRL have been deployed in real-world applications^5^. Examples include DINOv2^6^, OpenCLIP^7^ for vision, and GPT-4^8^ for free text. In the medical field, similar models like MedCLIP^9^ and MedSAM^10^ have also been developed, trained specifically with medical imaging and textual data. These models are trained on extensive datasets and are open-source, making them easily deployable. The representations they learn from the unlabeled data are designed for versatile use, enabling application across various downstream tasks, often referred to as foundation models^11,12^. By providing efficient learned representations, these models offer new opportunities to enhance the performance of existing models and reduce the need for large, manually annotated datasets.

Analyzing EHRs data poses several challenges, including its sparsity, high-dimensionality, and complex interrelationships^13^. EHRs consist of irregularly spaced visits over time, with each visit containing a subset of thousands of possible medical codes, along with laboratory test results, unstructured text, and images^14^. In this review, we focus specifically on EHR categorical data, also referred as structured data. EHR categorical data includes medical codes such as diagnoses, procedures, medications, and laboratory test codes. Categorical data is easier to de-identify following HIPAA guidelines^15^, enabling faster construction of large datasets, as it is generally considered safer in terms of patient privacy compared to clinical free text^16^.

**Fig. 1:**
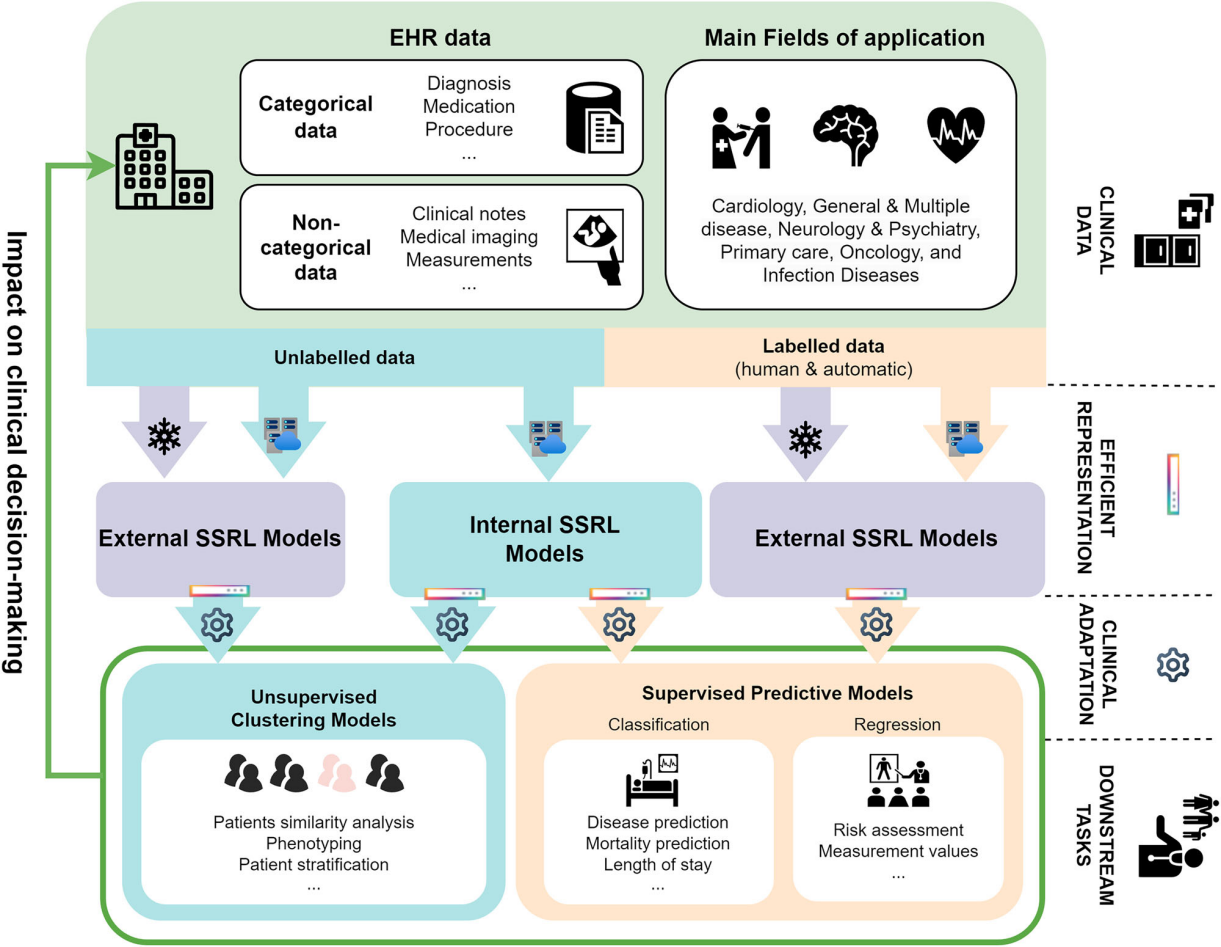
Description of medical application of SSRL in the clinical settings and the flow of data. EHR data follow a cyclical process, beginning at health centers, where they are either used to train internal SSRL models (blue box) or directly supplied to external SSRL models (purple box). These models convert the data into efficient representations, which are then adapted based on the specific downstream tasks. The results from these downstream tasks are sent back to the health centers, facilitating the delivery of effective medicine and medical knowledge discovery. Blue and orange arrows represent unsupervised and supervised learning tasks, respectively. For efficient representations, the snowflake and cluster icons stand for obtaining efficient representations with respectively frozen (only inference) or trainable (with high computational resources such as high-performance computing) SSRL models. The gear icon signifies the training of downstream models using moderate resources, such as multiple GPUs. The potential use of externally developed SSRL models is highlighted in purple.

SSRL in DNNs automatically discovers and extracts features from unlabeled data^11^. Unlike supervised learning, which relies on labeled datasets, SSRL algorithms are trained to predict part of the data from other parts, which could be incomplete, transformed, distorted, or corrupted. Essentially, the model learns to ’recover’ whole, parts of, or merely some features of its original input^17^. This enables SSRL to identify patterns and structures within unlabeled data, producing efficient representation vectors. These vectors, along with trained SSRL models, can be used for clustering similar data points, enhancing data visualization, or serving as inputs for subsequent predictive models. Figure 1 illustrates the application of SSRL in clinical settings. This framework offers several advantages: it reduces the need for extensive manual labeling, can be generalized across different tasks without requiring full model retraining, and often outperforms the models trained on a similar number of labeled datasets. As a result, using these representations and models optimizes manpower, computational resources, and model performance^11,17^.

Despite the progress in large models for images and text, there is still a notable absence of large models based on EHRs in real-world applications. Previous reviews, including those by Si et al.^18^, Amirahmadi et al.^19^, Oss Boll et al.^20^, and Hama et al.^21^, have covered both supervised and unsupervised methods across various data types. However, none have systematically analyzed representation learning using unlabeled EHR categorical data, covering both clustering and prediction tasks. As a result, the reader is left without a clear understanding of the current State-of-the-Art (SOTA) trends, limitations, and opportunities in this area. This review, covering studies from 2019 to 2024, addresses this critical gap by offering detailed insights into the latest SSRL methodologies for unlabeled EHR categorical data. We assess their potential applications, identify appropriate scenarios for their deployment, and evaluate the feasibility of implementation in current clinical settings. This review offers valuable guidance for future research, practical healthcare data analysis, and implementation in hospital settings. Our scoping review answers three main research questions: i) What techniques and models are used for analyzing categorical data? (see sections “Type of data”, “Data preprocessing” and “Self-supervised learning models” ii) How can SSRL models enhance clinical decision-making? (see sections “Fields of application” and “Evaluation tasks”) and iii) What are the current trends in the research field, and how do they impact medical settings? (see section “Discussion”). The detailed research questions and the full methodology of the scoping review are described in Methods section.

We differentiate our work from the narrative review by Wornow et al.^5^ by specifically addressing SSRL using unlabeled categorical data from EHRs, regardless of the changing definitions and usages of the term “foundation models” to provide the reader a systematic analysis and comprehensive view of current SOTA in the field. Notably, while we overlap with only 12 of the same papers over a similar time period, we include an additional 34 studies, underscoring the broader scope of our review. We highlight several agreements with their claims and systematically clarify the areas of similarity.

This scoping review is intended for an audience comprising medical professionals, data scientists, and healthcare stakeholders such as decision-makers and hospital IT teams. By synthesizing studies from databases across these fields, we aim to bridge the gap between clinical expertise and advanced data science techniques. Considering the societal and economic impact of leveraging recent research advances in SSRL, our goal is to provide valuable insights that enhance clinical decision-making processes, encourage interdisciplinary collaboration in healthcare informatics, and assist decision-makers in effectively adapting their IT infrastructure and data management strategies.

## Results

This section provides a comprehensive overview of the findings from our scoping review, organized around subsections that emerged during our analysis. We begin by outlining the characteristics of the included studies and the types of data utilized. Next, we examine the studies from the technical aspects including the data preprocessing techniques, SSRL model types, SSRL model comparison, models for downstream tasks, the evaluation metrics used, and the interpretability techniques. Finally, we analyze the studies from a clinical perspective focusing on the fields of clinical application, clinical downstream tasks, and the involvement of medical experts. Error! Reference source not found. summarizes the key features of the technical aspect, and Table 2 provides essential information on the studies from the medical perspective.

### Studies characteristics

**Fig. 2:**
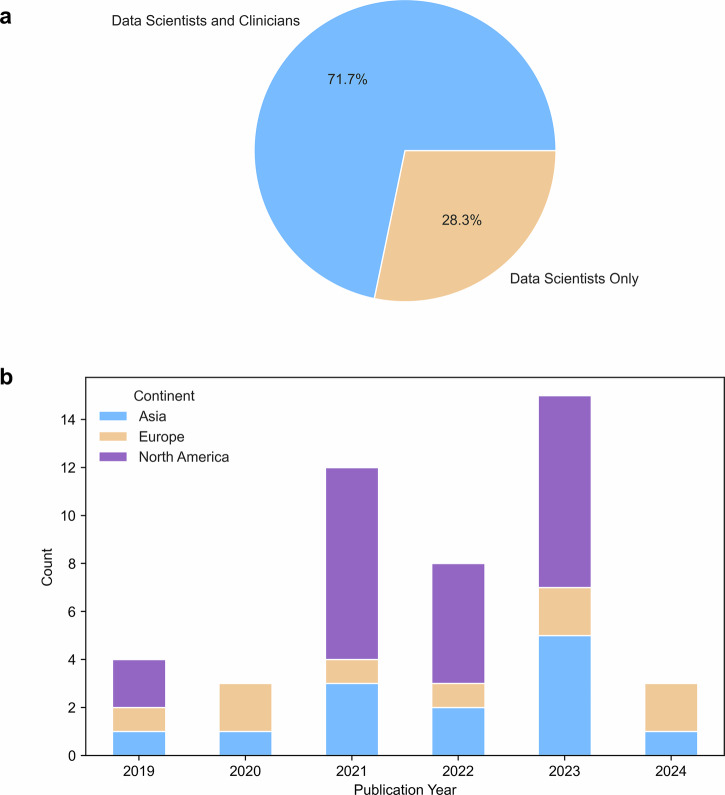
Meta-data from reviewed studies. **a** Composition of authors, categorized into two groups: those specializing in data science only, and those with expertise in both data science and medical fields. **b** Annual distribution of published studies from 2019 to 2024, categorized by continent.

As illustrated in Fig. 2, most of the research (*n* = 33, 72%) was conducted by interdisciplinary teams of medical experts and data scientists. The United States led in the number of published studies (*n* = 21, 46%), followed by China (*n* = 9, 20%) and the United Kingdom (*n* = 4, 9%). Despite this geographic diversity, only a few studies (*n* = 11, 24%) involved international collaborations. For details on the authors and research teams, refer to Supplementary Data 2

### Type of model and trend

Five main model types have been identified for representing EHR categorical data: Transformer-based models (*n* = 20, 43%), Autoencoder (AE) based models (*n* = 13, 28%), Graph Neural Network (GNN) based models (*n* = 8, 17%), Word-embedding models (*n* = 3, 7%), and Recurrent Neural Network (RNN) based models (*n* = 3, 7%). Studies that combine two or more model types are counted once for each corresponding model type. To assess their impact on research, we analyzed the number of citations for each model type.

**Fig. 3:**
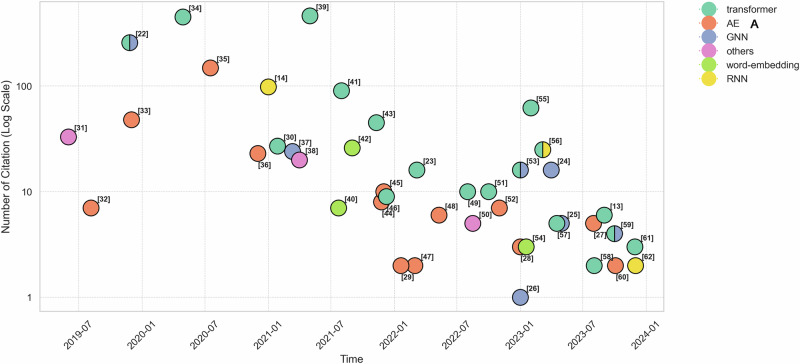
Number of citations for each study published from 2019 to 2023. Each data point represents a paper, including its corresponding reference and is color-coded by the model type used: Transformer, AE, GNN, Word-embedding, RNN, and others. Papers published in 2024 are not shown.

Figure 3 shows the papers published from January 2019 to December 2023, their citation counts by July 2024, and their corresponding model types. Based on the number of citations, Transformers, RNN, and GNN models are the most impactful, with Transformer models showing particularly high citation counts for papers published from 2020 to 2023.

### Type of data

Studies utilize various data types to represent patients and medical knowledge. Typically, patient representation is derived from EHRs, incorporating both categorical and non-categorical data. Additionally, external medical knowledge can be integrated into models through data collected beyond EHRs. For detailed information on the modalities used across studies, see Supplementary Data 3.

Among the categorical data types in EHRs, diagnosis codes are the most frequently used (*n* = 45, 98%), including ICD-9, ICD-10-CM, and SNOMED-CT. Medication codes (*n* = 32, 70%), such as ATC and SNOMED-CT, along with procedure codes (*n* = 20, 43%) like CPT and ICD-10-PCS. To enhance patient representation, non-categorical data may also be included. The most common non-categorical data types are patient age (*n* = 19, 41%), clinical measurement values (*n* = 15, 33%) such as BMI, heart rate, and systolic blood pressure, and clinical narratives from physicians and practitioners (*n* = 7, 15%).

The integration of external data sources can further enrich patient profiles. Medical knowledge graphs and ontologies provide rich hierarchical information, while medical text corpora contain expert medical knowledge. These external sources offer a comprehensive understanding of clinical concept interactions. Among external data sources, ontologies are the most used (*n* = 7, 15%), they are employed to obtain the medical concept embeddings^22–28^ and for SSRL training task^23^. Other significant external data sources include medical knowledge graph^25,29^ and medical text corpora^30^.

### Data preprocessing

Most models treat each data element as a distinct unit or token (*n* = 44, 95%). The identified data preprocessing techniques address various aspects such as numerical data, categorical data, data cleaning, and data shuffling. Some studies (*n* = 7, 15%) performed categorization by converting exact ages into intervals and clinical measurements into categories like high, normal, and low, based on clinical evaluation standards^31–37^. When maintaining the numerical nature of data, missing value imputation^30,38,39^ and value normalization^31,39–41^ have also been employed.

Some studies standardize data elements by mapping them to known ontologies^23,36,42,43^. A common approach to reduce dimensionality and data sparsity is using only the first digits of codes, effectively replacing them with parent nodes in the hierarchical ontology (*n* = 15, 33%).

In terms of data cleaning, typical practices include the removal of rare medical terms^14,32,37,42,44,45^ and the elimination of duplicated terms within a specific time range^22,42,46,47^. Additionally, shuffling the order of medical concepts within a time window^33,47^ was shown to help the model to generalize better, by mitigating the impact of arbitrary sequencing and emphasizing the importance of co-occurrence over specific order. This method can also be considered as a form of data augmentation. Detailed information on data preprocessing across studies can be found in Supplementary Data 4.

### Self-supervised learning models

There are two primary self-supervised learning training strategies: generative and contrastive. Generative tasks involve models predicting parts of the data from other parts, which may be incomplete, transformed, masked, or corrupted. These tasks, such as autoregressive prediction and masked modeling, help the model learn to recover whole or partial features of its original input^17,48^. Contrastive tasks, on the other hand, focus on distinguishing between similar and dissimilar data points, helping the model capture discriminative features that are essential for understanding different types of data^48^. Both task types are crucial for training models to generate rich, generalized representations from unlabeled data^48,49^, and they are applied across various model architectures. The objective of these models is to capture essential patterns and features in the data and output the learned representation which is typically a fixed-length, high-dimensional vector that condenses large amounts of information. Five major architecture types have been identified in the studies, each trained with unlabeled data with different training tasks. Details of the SSRL models used and the temporality monitored in each study are provided in Supplementary Data 5.

Transformer-based models are among the most impactful model types in the studies. In the medical domain, most transformer-based models treat patients as documents, visits as sentences, and medical concepts as tokens, capturing detailed patient histories. BERT^50^ is a transformer encoder-only model that effectively learns data representations by processing and contextualizing complex sequences of information. BERT models can be trained using various techniques, such as training with only Masked Language Model (MLM) by predicting randomly masked medical concepts in each EHR sequence^34,43,44,51–53^, enhancing its contextual understanding. Training both with MLM and auxiliary tasks^13,22,39,54–56^, further refine the model’s representations by guiding it with specific medical insights. Additionally, self-contrastive learning techniques help improve BERT’s robustness and accuracy in capturing meaningful patterns in medical data^30,35^. Other transformer-based training tasks include next visit code prediction^23,36,45,57^, medical code category prediction^23^, medication-diagnosis cross prediction^26^, and token replacement detection ELECTRA^58^.

AE-based models are encoder-decoder models that aim to reconstruct the input, enabling the learning of data representations in a compressed, lower-dimensional space. AEs are designed to learn the most salient features of the data, which can be particularly useful for capturing the underlying structure of categorical EHR data. Various deviations of AE were applied in the studies: Stacked Autoencoder^32,59^, Denoising Autoencoder^60^, Autoencoder with RNN units, such as GRU^31^ and LSTM^38,41,61–63^. Additionally, AE can be combined with other models such as collective matrix factorization^29^, CNN^42^, and clustering algorithms^27,64^.

GNN-based models use graph learning to represent medical ontologies, hospital visits, and disease co-occurrence. Nodes represent the medical concepts and personal entities, linked by edges indicating their relationships. Graph attention models were used to learn the medical concept embeddings within medical ontologies^22,26^, with these embeddings frequently serving as initializations for further model training. Random walk technique is used to embed doctors according to their specialty^65^. Graph contrastive learning^25,28^ generates multiple views of augmented hospital visit graphs by modifying the original graph with node or edge perturbations, allowing the model to learn robust representations by contrasting positive pairs against negative pairs. These approaches ensure that the learned embeddings accurately reflect the complex relationships inherent in medical data^49^.

Word-embedding-based models convert words into numerical vectors, allowing computers to understand their meanings and relationships from their context in a sequence of words. The model learns to map each word or concept to a dense vector representation, capturing semantic similarities based on co-occurrence patterns. Patient EHR data, composed of a sequence of medical concepts ordered by time, are used to train the representation model to predict medical concepts based on their surrounding context, helping the model to understand relationships between concepts. Various algorithms were identified, such as Glove^46^, Word2vec^33,46,47^ and FastText^46^.

RNN-based models are designed to capture temporal dependencies in sequential data, making them well-suited for tasks involving time-series EHR data. These models are trained with the objective of predicting future medical events based on a patient’s historical data. Studies^14,36,37^ use a specific type of RNN, GRU. The models were trained to predict the set of medical code of day *t* based on the medical codes of previous days. To enhance the temporality, these studies have also included the time gap information in the input.

### SSRL models comparison

Different self-supervised representation learning models offer unique advantages and face specific limitations. The choice of models depends on several factors, including the size of the available dataset, the importance of temporal modeling for the downstream tasks, and the computational resources available at the institution.

AEs excel at dimensionality reduction^66^ and are well-suited to relatively moderate datasets (average size: 166k in the included studies). However, they struggle with high-sparsity data^67^ and cannot inherently model temporal dependencies without incorporating sequential components, such as RNNs, CNNs, and Transformers.

Word embedding models are designed to map medical concepts or tokens into dense vector spaces that capture contextual information and syntactic relationships in the data^68^. They perform well with a moderate dataset (average size: 139k in the included studies). However, traditional word embeddings are static and fail to account for the temporality or the sequential order of the input data, necessitating their integration with sequential components.

GNNs perform well with small to moderate datasets (average size: 55k in the included studies) and are particularly effective at representing relational data, such as knowledge graphs, patient networks, and ontologies^69^. They offer strong interpretability by visualizing relational data, aligning with clinical knowledge. However, GNNs alone cannot fully address temporal dependencies, necessitating their integration with sequential components.

RNNs^70^ are well-suited for larger datasets (average size: 1.8 M in the included studies) and excel at capturing temporal patterns in sequential data. However, their training process is not parallelizable, leading to time inefficiencies^71^.

Transformers dominate SSRL research due to their ability to simultaneously capture long-range dependencies and temporal patterns^72^, offering scalability for large datasets and robust performance across diverse tasks. However, training these models from scratch necessitates substantial amounts of data (average size: 3 M in the included studies), and their high computational cost and complexity can pose significant challenges for deployment in resource-limited settings^73^.

### Downstream task models

Predictive models for classification are used with the trained SSRL model as their backbone, to which a specific classification head is added. These predictive models require labeled data for training on specific tasks. Among the articles that have mentioned the predictive models used for classification tasks, different model types have been identified. These models are predominantly characterized by simple architectures which are easy to train. Some studies employ shallow neural networks such as linear layer^23,39,44,57^, logistic regression (LR) (*n* = 8, 17%), and support vector machines (SVM)^31,74^. Models that can capture more complex data patterns such as feedforward neural networks (*n* = 12, 26%) and RNN^13,40,54,55,62,65^ (*n* = 6, 13%), are also applied.

Clustering and visualization models are used with the data representation vector as input. We identified several techniques employed across the literature. T-distributed Stochastic Neighbor Embedding (t-SNE) emerged as the most frequently used model for data representation visualization and cluster interpretation (*n* = 12, 26%). In terms of clustering techniques, K-means^33,38,47,62^ was found to be the most common method. These clustering models take the embedding vectors generated by trained representation learning models as input.

### Evaluation metrics

The evaluation of these tasks is primarily categorized into classification and clustering assessments, each employing different metrics to measure performance.

For classification tasks, the majority were binary, the most frequently used classification metric was AUROC (*n* = 21, 46%), followed by AUPRC (*n* = 14, 30%), accuracy (*n* = 10, 22%), and F1 (*n* = 9, 20%), while other metrics were also used but less frequently, such as precision (*n* = 6, 13%) and sensitivity (*n* = 5, 11%). A few studies have evaluated multi-class classification tasks. Metrics such as average precision^51,74^, precision at k^44,45^, macro-F1^29,65^ and weighted F1^24,29^ were each reported in the studies^10,24,28,74^.

For clustering tasks, despite the prevalence of clustering studies, only a few employed specific clustering analysis metrics. Silhouette analysis (*n* = 4, 9%) was the most frequently used metric, followed by Davies-Bouldin index^33,41^ (*n* = 2, 4%) and purity score^42,64^ (*n* = 2, 4%)

### Interpretability

Interpretability in machine learning is defined as the extraction of relevant knowledge from a machine-learning model concerning relationships either contained in data or learned by the model^75^. Attention weight analysis was used in several studies (*n* = 6, 13%). Statistical analysis of the clusters was employed in some papers (*n* = 3, 6%). For post-hoc interpretability, methods such as Integrated gradient^13^ and Gradient-based saliency^45^ were utilized. Most of the papers interpreted their results using visualization computed by t-SNE (*n* = 12, 26%) and Uniform Manifold Approximation and Projection for Dimension Reduction (*n* = 3, 6%). Ten papers involved medical expert interpretation. Overall, only two papers attempted post-hoc interpretability methods on trained models. Refer to Supplementary Data 7 for detailed information on the interpretability methods used in the studies.

### Fields of application

Our scoping review identified various tasks across the articles. These tasks were distributed across various clinical domains, with Cardiology^24,31,32,34,35,40,41,43,53–56,60,61,74^ (*n* = 15, 33%), both General & multiple diseases (*n* = 11, 24%), Neurology & Psychiatry and Primary Care (*n* = 9, 20%) being the most frequently studied areas. Oncology (*n* = 6, 13%), followed, while Infectious Diseases^39,40,47,61^, Endocrinology^35,38,42,60^ and Respiratory^13,23,32,64^ each had 4 downstream tasks (*n* = 4, 9%). Gastroenterology^40,42^ and Nephrology^27,35^ had the lowest number of downstream tasks (*n* = 2, 4%). A detailed overview of the clinical events and their corresponding clinical domain mapping can be found in Supplementary Data 1.

### Evaluation tasks

Upon training, deep learning models have developed an intrinsic representation of the data, which can be general, supporting multiple tasks, or task-specific, focusing on a single or a few similar tasks. Representation quality is evaluated in various clinical tasks, including predictive tasks, or patient phenotyping. For detailed information on the evaluation tasks in the studies, see Supplementary Data 7.

Among the 73 predictive tasks, the primary focus was on disease prediction (*n* = 27, 59%), followed by mortality prediction (*n* = 11, 24%), readmission prediction^14,26,28,32,36,53,55,65,76^ (*n* = 9, 20%), hospitalization (*n* = 5, 11%), and length of stay prediction (*n* = 4, 9%). In addition to these, other tasks included medication recommendations^22,26,40^ (*n* = 3, 7%), ICD coding^56^, doctor recommendations^65^, ICU transfers^14^, emergency department visits^63^, and high medical resource utilization^63^.

Beyond predictive modeling, patient phenotyping plays a crucial role in understanding patient populations. Of the 33 patient phenotyping tasks, clustering was primarily used for visualization (*n* = 15, 33%), patient similarity assessment (*n* = 8, 24%), characterization of clusters (*n* = 3, 9%), patient subtyping (*n* = 2, 6%), and patient stratification (*n* = 1, 3%).

### Medical expert involvement

Medical experts were involved across different stages of the studies, with varying degrees of participation. Among the reviewed publications, expert participation was the most prominent were study design (*n* = 14, 30%) and result interpretation (*n* = 14, 30%). Feature selection also saw substantial expert input (*n* = 10, 22%), while dataset extraction had more limited expert participation (*n* = 4, 9%).

## Discussion

The most employed SSRL model types include Transformer-based, AE-based, and GNN-based architectures. These models, often referred to as foundation models, are trained to reconstruct or predict corrupted portions of input data^11^. The core strength of SSRL is the ability to construct a vectorized database, where clinical data, such as patient or encounter information, is embedded directly into low-dimensional representation embeddings. These embeddings can be easily retrieved and used for various medical ML research and applications, such as predictive modeling, personalized medicine, and disease prognosis, as shown in Table 2. To train such SSRL, it is advised to use a broader patient cohort, then transfer learned information from the entire patient population to specific models relevant to a subset of the population^13,14^. The average unlabeled dataset size used for training SSRL models is 1.3 million data elements, compared to 96k data elements for labeled datasets used in downstream tasks, see Table 1 and Supplementary Data 6 for detailed information on SSRL training cohort selection, types of cohorts, and cohort size. This comprehensive data exposure enhances the models’ ability to learn underlying medical knowledge, thus improves predictive performance with specific patient subsets and even generalizes to other external datasets^23^.

**Table 1:** Technical overview of studies

		Dataset										Model type						Task type		
		categorical				Numerical		Patient number		Dataset type										
Model name	Year	Procedure	Diagnosis	Medication	Lab. tests	Age	Measurement	Unlabeled	Labeled	Private	Public	AE	Transformer	GNN	RNN	Word embedding	Others	Classification	Clustering	Regression
Liang et al.^74^	2019		x	x	x			<1k	<1k	x							a	x		
de Lusignan et al.^59^	2019		x			x		11k		x		x							x	
G-BERT^22^	2019		x	x				83k	6k		1		x	x				x		
Ruan et al.^31^	2019		x	x	x	x	x	5k	5k	x		x						x	x	
BEHRT^51^	2020		x			x		1.6M	700k	x			x					x	x	
ConvAE^42^	2020	x	x	x	x			1.6M		x		x							x	
Enhanced Reg^32^	2020		x		x	x	x	104.4k	73k	x		x						x		
CLMBR^14^	2021	x	x	x	x	x		3.4M	131k	x					x			x		
EDisease^30^	2021		x		x	x		1M	816k	x			x					x	x	
ME2Vec^65^	2021	x	x	x				111k	11k	x	2			x				x	x	
PLGMNN^40^	2021			x	x		x	<1k	<1k	x	1						b	x		
Med-BERT^54^	2021		x					28.4M	43k	x			x					x		
Huang et al.^33^	2021	x	x	x	x	x	x	105k		x						x			x	
BRLTM^52^	2021	x	x	x		x		44k	10k	x			x					x		
Phe2vec^46^	2021	x	x	x	x			300k		x						x			x	
CEHR-BERT^55^	2021	x	x	x				2.4M	591k	x			x					x	x	
DICE^41^	2021		x	x	x	x	x	1k	1k	x		x						x	x	
Chushig-Muzo et al.^60^	2021		x	x				6,5k		x		x							x	
Poulain et al.^34^	2021		x	x			x	7k			3		x							x
Kumar et al.^29^	2022	x	x	x	x			29k	29k		1	x						x		
Shao et al.^64^	2022		x	x	x			30k		x		x							x	
Claim-PT^23^	2022	x	x	x		x		1.9M	1k	x			x					x		
Navaz et al.^61^	2022		x			x	x	5k			4,5	x						x	x	
CEHR-GAN-BERT^56^	2022	x	x	x		x		55k	<1k		3,6		x					x		
CEF-CL^82^	2022	x	x					48k	48k	x	3						c	x		
ADADIAG^43^	2022		x		x			28k	6k	x	6		x					x	x	
Manzini et al.^38^	2022		x	x		x	x	11k		x		x							x	
Herp et al.^62^	2023	x	x	x				19k	19k	x		x						x	x	
MMMGCL^28^	2023	x	x		x			14k	4k		1,2			x				x		
MedM-PLM^26^	2023		x	x				40k	5k		1		x	x				x		
Ta et al.^47^	2023	x	x	x	x		x	11k		x						x			x	
Hi-BEHRT^35^	2023	x	x	x	x	x	x	2.8M	406k	x			x					x		
CLMBR-2^36^	2023	x	x	x	x		x	1.8M	157k	x			x		x			x		
Sherbet^24^	2023		x					46k	7k		1,2			x				x		
Ru et al.^53^	2023	x	x	x	x			299k	31k	x			x					x		
SeqCare^25^	2023	x	x	x	x			14k	2k	x	1			x				x		
Liu et al.^27^	2023		x					2k			1	x							x	
IPDM^58^	2023		x					119k	24k		1,7		x					x	x	
ExMed-BERT^13^	2023		x	x	x	x	x	3.5M	80k	x			x					x		
Pellegrini et al.^39^	2023		x	x	x	x	x	22k	22k		1,8,9		x	x				x	x	
Jones et al.^63^	2023		x	x				27k	11k	x		x						x		
TransformEHR^57^	2023		x			x		6.5M	10k	x			x					x		
CLMBR-3^37^	2023	x	x	x			x	242k	18k	x					x			x		
Profile model^44^	2024		x			x		1M	53k	x			x					x	x	
Seki et al.^76^	2024		x	x	x		x	32k	15k	x				x				x	x	
Foresight^45^	2024	x	x	x		x		710k	37k	x			x					x		

A summary of the published year, data type, patient number, model type, and task type of the studies. Other model type: a: Deep belief network, b: local-global memory neural network, c: contrastive learning. Public dataset: 1: MIMIC-III, 2: eICU, 3: All of Us Program, 4: epidemiological COVID-19 data, 5: Framingham offspring heart study, 6: MIMIC-IV, 7: Alzheimer’s Disease Neuroimaging Initiative, 8: TADPOLE, 9: Sepsis Prediction Dataset.

Labeling EHR data is manpower-intensive and time-consuming. SSRL models, especially those designed as general-purpose foundation models, streamline the development process by eliminating the need for labeled data or task-specific training^5^. As shown in Table 2, over half of the studies (*n* = 24, 52%) focused on general-purpose SSRL models, which, once trained, can be reused across various end tasks, in contrast to the task-specific nature of supervised learning models. For clinical downstream tasks, the integration of SSRL models improves the model’s predictive performance. Studies have shown that SSRL models, when trained on large unlabeled datasets, can improve predictive performance in fine-tuned settings, often requiring less labeled data compared to traditional supervised models^35,55^.

**Table 2:** Clinical overview of studies

	Medical domain						Interpretability					Evaluation							
												Downstream tasks							
model name	Cardiology	General & multiple diseases	Neurology & Psychiatry	Primary Care	Oncology	Other domains	Attention analysis	Cluster analysis	Post-hoc interpretability	Embedding visualization	Medical expert interpretation	Disease prediction	Mortality prediction	Readmission prediction	Length of stay prediction	Patient similarity	Hospitalization	Other tasks	External evaluation
Liang et al.^74^	x					a						m							
de Lusignan et al.^59^				x							x							8	
G-BERT^22^				x														1	
Ruan et al.^31^	x									x		m	x						
BEHRT^51^		x					x			x	x	m						8	
ConvAE^42^			x		x	d		x		x	x							9	
Enhanced Reg^32^	x					c						x		x					
CLMBR^14^		x											x	x	x			2	
EDisease^30^				x						x	x		x			x	x		x
ME2Vec^65^				x	x					x		m		x				4	
PLGMNN^40^	x				x	b,d												1, 10	
Med-BERT^54^	x				x		x				x	x							x
Huang et al.^33^			x							x						x			
BRLTM^52^			x				x					x							
Phe2vec^46^		x								x	x					x			
CEHR-BERT^55^	x	x										x	x	x			x		
DICE^41^	x							x		x	x	m				x			
Chushig-Muzo et al.^60^	x					a												5	
Poulain et al.^34^	x																	11	
Kumar et al.^29^				x								m	x						
Shao et al.^64^						c		x		x	x					x			
Claim-PT^23^			x			c						x							x
Navaz et al.^61^	x					b							x			x			
CEHR-GAN-BERT^56^	x			x								x	x						
CEF-CL^82^		x										x							
ADADIAG^43^	x						x			x		x							x
Manzini et al.^38^						a					x							5	
Herp et al.^62^					x							m						10	
MMMGCL^28^				x									x	x	x				
MedM-PLM^26^				x			x							x				1, 3	
Ta et al.^47^						b					x							5	
Hi-BEHRT^35^	x		x			a,e						x							
CLMBR-2^36^		x											x	x	x		x		
Sherbet^24^	x			x								m					x		
Ru et al.^53^	x													x					
SeqCare^25^		x										m							
Liu et al.^27^						e				x								8	
IPDM^58^			x							x		m	x					10	
ExMed-BERT^13^						c			x		x	x							x
Pellegrini et al.^39^			x			b	x			x		m	x		x				x
Jones et al.^63^			x									x					x	6, 7	
TransformEHR^57^			x		x						x	x							x
CLMBR-3^37^		x									x	x							x
Profile model^44^		x								x		m							x
Seki et al.^76^		x								x				x					
Foresight^45^		x							x		x	m							

A summary of the medical domain, interpretability, evaluation tasks, and model external data evaluation of the selected studies- Other medical domain: a: Endocrinology, b: Infectious Diseases, c: Respiratory, d: Gastroenterology, e: Nephrology. Other evaluation tasks: 1: medication recommendation, 2: ICU transfers, 3: ICD coding, 4: doctor recommendation, 5: patient subtyping, 6: emergency department visit, 7: high medical resource utilization, 8: characterization of clusters, 9: patient stratification, 10: prognosis analysis, 11: multiregression, m: multilabel.

**Fig. 4:**
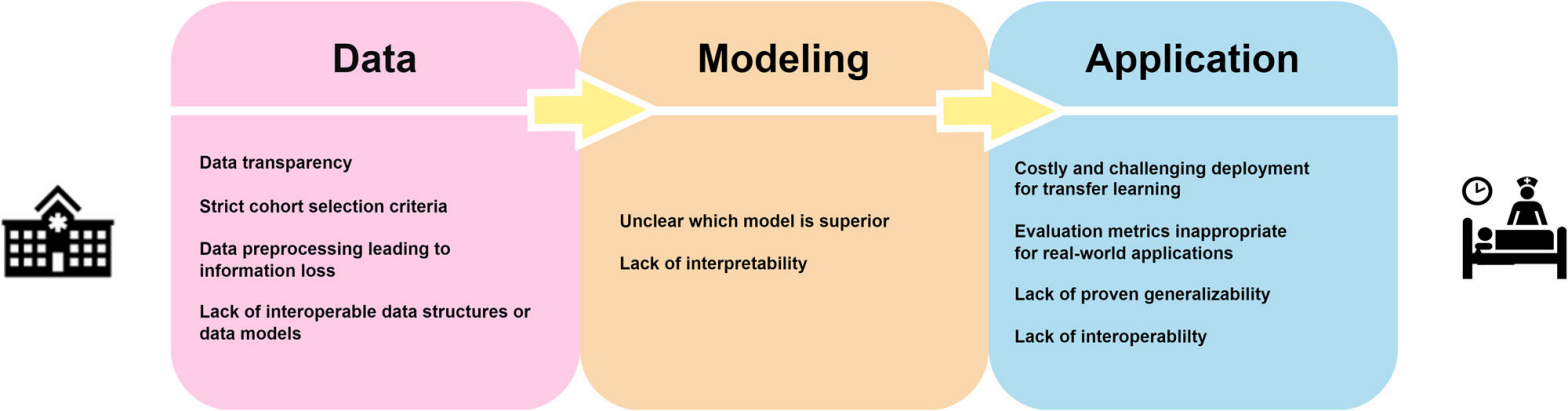
Summarization of limitations in current research. Key challenges across three stages: Data, Modeling, and Application. Data-related issues include transparency, strict cohort selection, preprocessing-related information loss, and lack of interoperability. In modeling, challenges stem from uncertainty in model superiority and poor interpretability. Application limitations include costly deployment, inadequate evaluation metrics, lack of generalizability, and interoperability barriers.

Despite these advantages, several challenges persist in the current research landscape, compassing data, modeling, and real-world application, as illustrated in Fig. 4. One of the primary concerns is data. Due to different clinical practices and economic reasons, datasets collected from different regions may differ a lot, which is called data shift. Most studies (*n* = 26, 57%) rely solely on private data collected from their medical sites. Only a few studies demonstrate model generalizability and transparency using public datasets (*n* = 11, 24%) or a combination of public and private datasets (*n* = 9, 20%). Additionally, there is a notable lack of public EHR dataset resources. The most frequently used datasets are MIMIC-III, MIMIC-IV, and eICU, which focus on intensive care data, whereas public datasets for general wards are lacking. See Table 1 and Supplementary Data 2 for the datasets used and their availability information.

Cohort selection introduces further challenges, often leading to selection bias. Rigorous cohort selection criteria, while ensuring data relevance and quality, can result in unrepresentative patient samples, thus affecting the generalizability of the findings^14^. Studies frequently exclude patients based on the number of visits, medical codes, age range, and specific medical conditions, leading to cohorts that may not reflect the realistic patient population and often include more severe cases^22,23^.

Furthermore, expert knowledge is rarely integrated into dataset construction. Only 9% of studies reported using domain experts in defining patient cohorts. This lack of clinical input raises concerns about whether datasets reflect real-world patient diversity and clinical complexity.

Data oversimplification is a common practice, where numerical data is categorized, and medical codes are truncated, which, while reducing input data dimensionality, introduces significant information loss and potential biases^77^. For example, reducing ICD-9 codes to their first three digits decreases the number of concepts from 9285 to 1131^52^, resulting in a loss of granularity and potentially important clinical details, Detailed information on the impact of preprocessing on the number of features across studies can be found in Supplementary Data 4.

Finally, the choice of coding systems, particularly the use of ICD for EHR analysis, raises concerns. ICD coding is often influenced by billing requirements rather than clinical accuracy, leading to potential biases. Additionally, since there is no unique mapping of a physician’s diagnosis to a coding scheme such as ICD, there is a tendency to select the code that delivers the greatest economic benefit from among several possible codes^13^. ICD-9, despite being the most frequently used ontology in these studies, has limited clinical relevance, as it does not cover all health conditions^64^. Moreover, variations in ICD coding across countries complicate transfer learning and hinder the development of universally applicable models^61^.

**Fig. 5:**
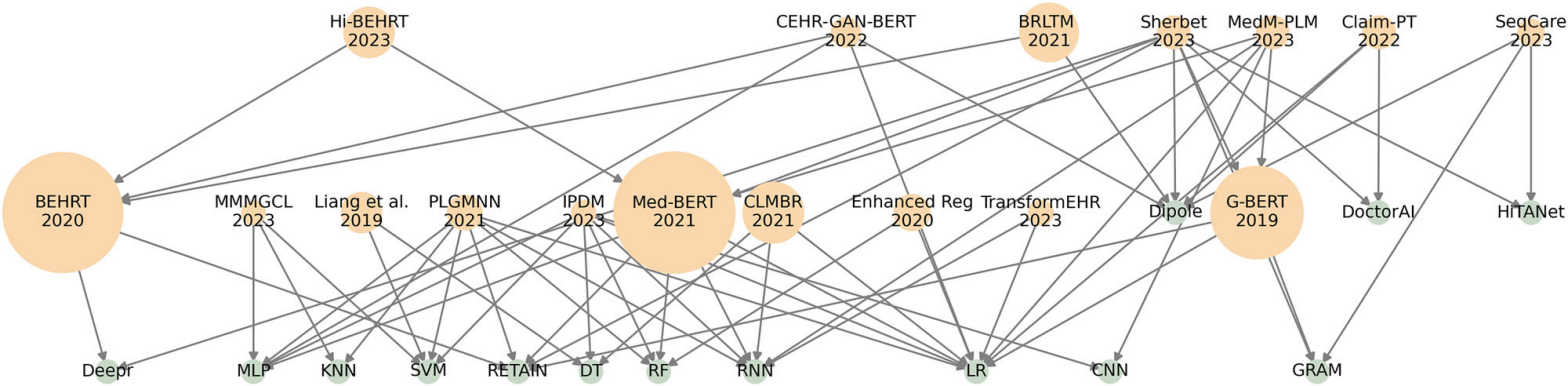
Comparison of performance between different models. SSRL models included in the review (yellow) and supervised learning models (green). The size of the yellow dots scale with the number of citations. Arrows indicate the comparison direction, pointing from the comparator to the comparand.

From a modeling perspective, most studies were evaluated using predictive tasks, typically comparing their model performance to classic end-to-end machine learning algorithms such as RNN, LR, SVM, and MLP, as shown in Fig. 5. On one hand, this demonstrates the superiority of SSRL frameworks over classic supervised learning ML baseline models. However, it also reveals a lack of direct comparison between different SSRL models. Additionally, the variety of clinical predictive tasks and datasets used makes it challenging to determine which model is optimal for a given task. Nonetheless, we observed that recent studies increasingly benchmark their models against other SSRL frameworks.

Another limitation of the modeling is the lack of interpretability. Deep learning models are often considered black boxes and can suffer from hallucinations. In real-world medical applications, clinical reasoning and model interpretation are crucial for providing justifiable guidance in decision-making^11^. While most studies attempt to interpret the model outcomes based on attention weights, visual evaluation of clusters with t-SNE, and manual inspection, these interpretation methods can be subjective^78,79^. Only a few articles perform formal post-hoc interpretation. The lack of unbiased interpretation may reduce the credibility of the findings.

Beyond data and modeling concerns, the real-world adoption of SSRL models faces significant hurdles. Several key limitations hinder the deployment of SSRL models in practice. First, the evaluation metrics commonly used in research are often technically focused and may not align with specific clinical needs, as noted by Wornow et al.^5^. Medical datasets frequently exhibit a marked class imbalance, with a much higher prevalence of healthy cases compared to disease cases. In such scenarios, achieving high sensitivity is often more critical than high specificity, as missing a true positive case can lead to severe consequences. The reliance on conventional data science metrics could result in unforeseen outcomes when applied to clinical practice. Furthermore, despite the potential advantages of transfer learning, SSRL models are typically data-intensive, making it challenging to train such frameworks in environments with limited data, such as small hospitals. To benefit from state-of-the-art models, these institutions would need access to pre-trained models built on large external datasets. However, shareable pre-trained models are often unavailable due to data security concerns. Even when such models are available, there is evidence in only a few studies of the generalizability across diverse populations and clinical environments, with further research needed to establish broader applicability. As shown in Table 2, only nine studies have demonstrated the effectiveness of pre-trained models on external clinical datasets, highlighting a significant issue: the lack of proven generalizability of transfer learning across diverse populations and clinical environments. Furthermore, they may not be easily usable due to incompatibilities with different EHR coding systems, leading to interoperability challenges^23,43,80^. Thieme et al.^81^ explored challenges and provided recommendations based on real-world implementation experiences. However, their work does not specifically address SSRL deployment. To date, no comprehensive study has examined these factors holistically, and none of the included papers in our review explicitly mentions actionable recommendations for SSRL implementation.

The adoption of SSRL models in clinical settings depends on the specific characteristics of the dataset, the availability of annotated data, the nature of the downstream tasks, and the computational resources. In clinical settings with low cardinality datasets and abundant annotated data for specific tasks, traditional supervised learning approaches may remain more effective than SSRL. However, in resource-limited clinical settings, where data and computational resources are scarce, SSRL models can present an efficient alternative, depending on specific task requirements and data characteristics. Autoencoders, GNNs, and word-embedding models efficiently learn compact representations when temporality is not a concern. In cases where the sequential order of medical history is critical and no pretrained model is available, RNNs are a viable option. If a pretrained transformer model is accessible, it is generally the preferred choice due to its ability to leverage rich, contextualized representations from pretraining. Techniques such as Inference using a frozen architecture^31,32,37,82^, fine-tuning^83^, domain adaptation^43,80^, prompt engineering^84^ and continual pretraining^80^ can significantly reduce the need for extensive computational resources and annotated data, even when the data distribution differs from the pretraining dataset.

As SSRL models continue to evolve, researchers are exploring ways to improve their generalizability. One emerging trend is the development of publicly shared Foundational Models (FMs), inspired by advancements in Natural Language Processing (NLP). These models, trained on diverse datasets, have the potential to improve knowledge transfer across different clinical settings^23,85^. These FMs could be particularly beneficial for smaller healthcare institutions with limited private data and computational resources^39^. For example, collaborative studies between the United States and Austrian hospitals^13^, the application of models trained on adult data to pediatric cases^37^, and the transfer learning of models from EHR to insurance data^44^ demonstrate the versatility of FMs. A recent multi-center study highlighted the adaptability of a shared foundation model^80^, showing that continual training on local data required fewer than 1% of training examples to match the performance of fully trained gradient boosting machines (GBMs). This approach was 60% to 90% more sample-efficient compared to training a local FM from scratch, underscoring its feasibility for resource-limited settings. These advancements highlight the potential of FMs to bridge the gap between research and real-world clinical applications, enabling resource-limited institutions to leverage state-of-the-art models without the need for extensive local data or computational infrastructure.

For institutions with access to extensive data and computational resources, pretraining a model from scratch offers significant advantages. For example, the Med-BERT^54^ model, pretrained on data from 28 million patients, exemplifies the potential of SSRL in such scenarios. A pretraining phase lasting approximately one week on a high-performance GPU, costing approximately 11,000$ (see Supplementary Data 8), can produce a robust representation model, capable of understanding and predicting complex health outcomes. This approach has demonstrated improved performance on predictive tasks and transferability across various clinical datasets^24^. Pretraining from scratch is particularly beneficial when existing pretrained models do not align well with the institution’s data or task requirements. By leveraging their vast datasets, institutions can create highly customized models that outperform generic, off-the-shelf solutions. This makes in-house development a cost-effective and scalable strategy for large healthcare organizations aiming to harness the full potential of their data.

Given these challenges, future research should focus on three key areas: (1) improving data availability and standardization, (2) developing better benchmarking practices, and (3) fostering multi-institutional collaboration to enhance model generalizability.

Expanding and sharing public datasets is essential to improve the generalizability and robustness of SSRL models. Increasing the availability of public EHR datasets that cover a broader spectrum of medical care beyond intensive care units is crucial. Collaborative efforts among medical institutions, government agencies, and research organizations can facilitate this expansion. Additionally, establishing data-sharing agreements and frameworks that address privacy and security concerns will enhance model generalizability and transparency. Incorporating data from diverse populations and clinical settings will make models robust to data shifts^43,56,86^, enabling their application in small hospitals with domain adaptations. Medical data standardization is another key factor, as it improves interoperability across institutions. Recently, Guo et al.^80^ proposed to use the widely adopted Observational Medical Outcomes Partnership Common Data Model (OMOP CDM) to standardize the data integration, Ruth et al.^87^ demonstrated that unifying different medical vocabularies into a cohesive knowledge graph significantly enhances the integration and generalizability of clinical AI models. Revisiting coding systems to ensure clinical relevance and consistency across regions, such as adopting or developing comprehensive ontologies or knowledge graphs like SNOMED-CT^88^ or PheKG^87^, is also recommended. Researchers should also focus on avoiding excessive data simplification and categorization by exploring advanced techniques for handling high-dimensional data without significant information loss. This will ensure that critical clinical details are preserved, improving the accuracy and applicability of SSRL models in real-world settings.

Benchmarking different SSRL models using standardized clinical predictive tasks and datasets is another critical area for future research^85^. Enhancing interpretability is also critical. Developing transparent models or robust post-hoc interpretation methods, such as model-agnostic interpretability techniques and explainable AI (XAI) frameworks, will make models more clinically useful^89^. Recently Self-Explainable Models (SEMs) have shown great explainability by proposing meaningful concepts to the user in medical applications while keeping strong performance^90^. Collaborating with clinicians to interpret outcomes and validate findings will enhance credibility and relevance. Furthermore, demonstrating the possibility of models with minimal effort using advanced techniques like Low-Rank Adaptation (LoRA)^91^ and Retrieval-Augmented Generation (RAG)^92^ will make these models more adaptable and practical for real-world applications^13^.

Extensive validation across diverse populations and clinical settings is necessary to ensure the real-world applicability of SSRL models. Models must be tested on external datasets to prove their generalizability^80^. Additionally, adopting evaluation metrics that reflect clinical outcomes and practical utility, rather than relying solely on technical performance metrics, is also essential. Engaging clinicians in the evaluation process will ensure the metrics used are aligned with clinical needs and that the models provide actionable insights. Finally, evaluating the information loss during preprocessing should be a priority, with preprocessing methods adapted to the precise clinical use to preserve critical information.

Multicenter collaboration is another priority for advancing SSRL models in healthcare. Most studies in the EHR domain rely predominantly on private datasets, which, despite their data size, represent only a small fraction of global patient data^85^. This lack of diversity limits the generalizability of trained models. To address this limitation, multicenter collaboration should be encouraged. Such collaborations are particularly beneficial for tasks that are less dependent on the local institutes’ clinical practices^93^, such as disease patterns relationship discovery^87^, genetic factors identification^94^ and rare disease analysis^86^.

Collaboration can take various forms, including data-sharing initiatives^95^ and federated learning (FL). Data-sharing initiatives, which leverage extensive and diverse datasets across institutions, can improve model robustness and applicability. However, achieving data-sharing requires addressing data privacy concerns and building trust in AI systems through transparent and ethical guidelines. In contrast, FL allows institutions to train models without directly exchanging data, ensuring privacy and security^86,96^. This approach is particularly valuable in healthcare, where data sensitivity is a major concern.

Despite these advantages, multicenter collaboration faces several challenges. First, multicenter data has high heterogeneity, which requires harmonizing medical vocabularies and standardizing data model^87,97^ to ensure interoperability across institutions. Second, the data heterogeneity may result in a global optimal solution that may not be optimal for an individual local participant. To address this issue, some authors propose that an agreed definition of model training optimality should be established among all participants before the collaboration^86^.However, this potential limitation was not addressed in the analyzed studies, and further evidence is needed to assess the use cases for which multicentric federated models provide a clear advantage.

## Methods

### Study design and search strategy

We conducted a scoping review following the PRISMA extension for Scoping Reviews (PRISMA-ScR) guidelines^98^. To encompass both healthcare and engineering perspectives, we systematically searched five electronic databases: PubMed, MEDLINE, Embase, ACM, and Web of Science. The search was limited to papers published between January 2019 and April 2024.

Our search strategy was designed to identify studies meeting three criteria: (1) utilization of deep learning or neural networks, (2) application of un/self-supervised deep representation learning, and (3) use of electronic health records (EHRs) categorical data as the primary data source for SSRL model training. The search query combined the following keywords: (“deep learning” OR “neural network” OR “machine learning”) AND (“unsupervised” OR “self-supervised” OR “pretrain*” OR “pre-train*” OR “BERT”) AND (“electronic health record?” OR “ehr” OR “electronic medical record?” OR “emr” OR “Electronic Health Records” OR “health care data” OR “patient longitudinal” OR “patient trajectory”).

### Study selection

**Fig. 6:**
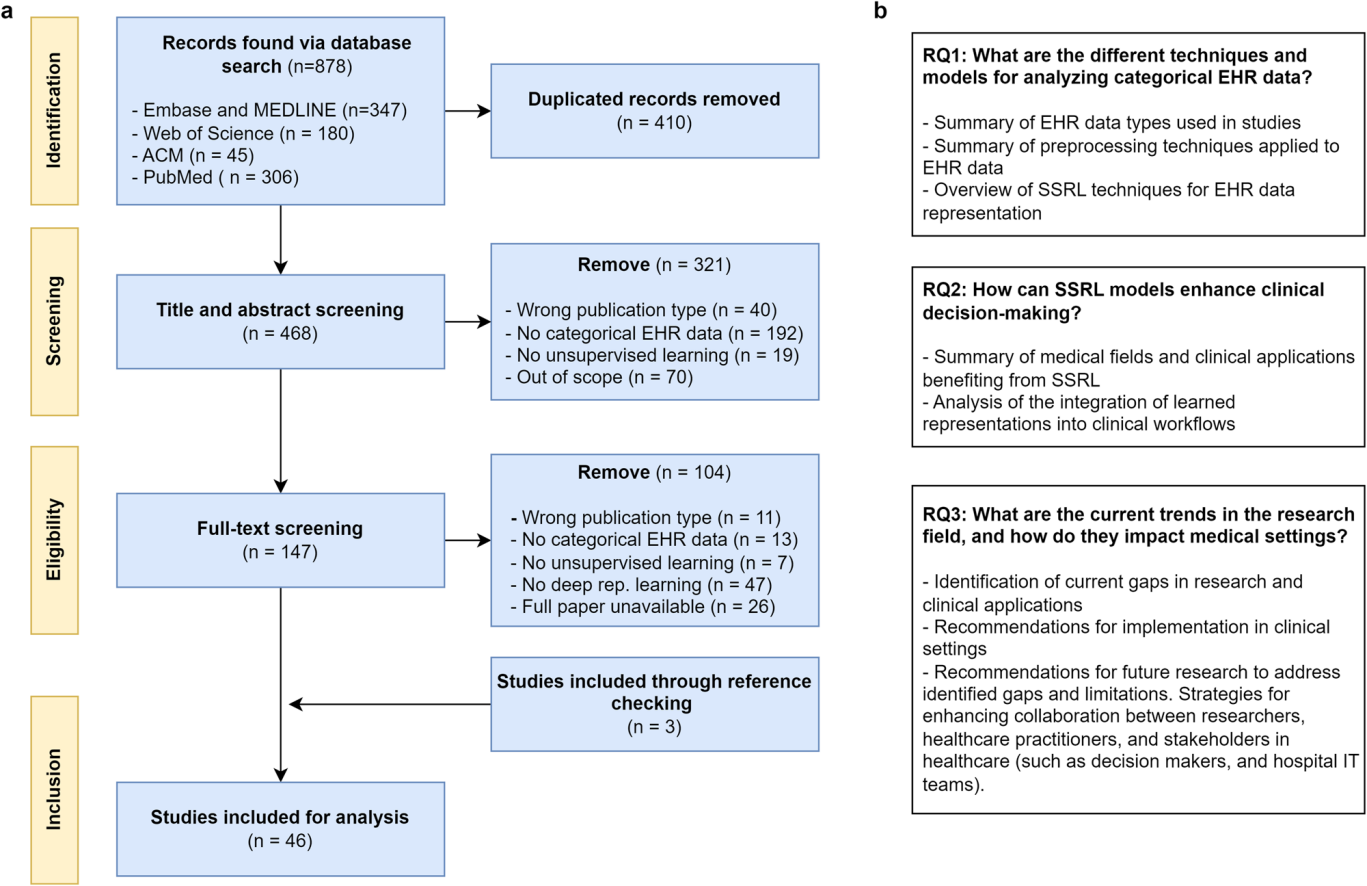
Overview of our PRISMA process and research questions of the review. **a** Flow diagram illustrating the PRISMA approach for the identification, screening, and selection of studies. **b** Research questions posed.

The screening process was conducted in multiple stages, see Fig. 6. First, a pilot screening of 100 papers was performed to refine the inclusion and exclusion criteria. Once consensus was reached, two independent reviewers screened all papers by title and abstract. The inter-rater reliability for the title and abstract screening process was 87%. Disagreements were resolved through discussion to achieve consensus. This was followed by a full-text review. We excluded studies that had duplicate titles, were review articles, did not use unsupervised deep learning on EHR categorical data for patient or encounter representation learning, or had outcomes not directly related to clinical decision-making. Studies focusing solely on physiological signals, clinical free texts, medical images, or clustering were also excluded. Three additional papers were identified through reference screening of included studies, resulting in a final sample of 46 papers for analysis.

### Data extraction and analysis

We extracted data on article information, authorship details, clinical data characteristics, unsupervised components of deep learning models, evaluation metrics, end tasks, as well as interpretability and transferability properties. A detailed description of these data items can be found in Supplementary Table 1. This information was compiled into a standardized spreadsheet and available in Supplementary Data 1–8, which was pre-tested by the team to ensure consistency. Two reviewers independently extracted the data, and discrepancies were resolved through discussion. Data analysis was performed using Python, primarily employing the pandas library for descriptive statistical techniques.

## Supplementary information

Supplementary Information

Supplementary Data 1

Supplementary Data 2

Supplementary Data 3

Supplementary Data 4

Supplementary Data 5

Supplementary Data 6

Supplementary Data 7

Supplementary Data 8

## Data Availability

All data generated or analyzed during this study are provided in the main article and Supplementary Data.

## Abbreviations

EHR: Electronic Health Record
SSRL: Self-Supervised Representation Learning
DNNs: Deep Neural Networks
SOTA: State-of-the-Art
RNN: Recurrent Neural Network
LSTM: Long Short Term Memory
GRU: Gated Recurrent Unit
BERT: Bidirectional Encoder Representations from Transformer
GNNs: Graph Neural Networks
AE: Autoencoder
MLM: Masked Language Model
CNN: Convolutional Neural Network
t-SNE: T-distributed Stochastic Neighbor Embedding
AUROC: the Area Under the Receiver Operating Characteristic Curve
AUPRC: Area Under the Precision-Recall Curve
NLP: Natural Language Processing
FL: Federated Learning. XAI Explainable AI
SEMs: Self-Explainable Models
LoRA: Low-Rank Adaptation
RAG: Retrieval-Augmented Generation
OMOP CDM: Observational Medical Outcomes Partnership Common Data Model
LR: Logistic Regression
SVM: support vector machine
GBMs: gradient boosting machines

## Code availability

This study did not involve the utilization of any custom code or mathematical algorithm.
