# Author Correction: A scoping review of self-supervised representation learning for clinical decision making using EHR categorical data

**DOI:** 10.1038/s41746-025-01919-1

**Published:** 2025-08-18

**Authors:** Yuanyuan Zheng, Adel Bensahla, Mina Bjelogrlic, Jamil Zaghir, Hugues Turbe, Lydie Bednarczyk, Christophe Gaudet-Blavignac, Julien Ehrsam, Stéphane Marchand-Maillet, Christian Lovis

**Affiliations:** 1 Division of Medical Information Sciences, Geneva University Hospitals, Geneva, Switzerland; 2 Department of Radiology and Medical Informatics, University of Geneva, Geneva, Switzerland; 3 Department of Computer Science, University of Geneva, Geneva, Switzerland

**Keywords:** Computer science, Machine learning, Predictive medicine

Correction to: *npj Digital Medicine* 10.1038/s41746-025-01692-1, published online 14 June 2025

In the original version of this article, the given and family names of authors were incorrectly structured. The original article has been corrected.

