# Supplementary material for: A scoping review of self-supervised representation learning for clinical decision making using EHR categorical data
Source: NPJ Digit Med. 2025 Jun 14;8:362. doi: 10.1038/s41746-025-01692-1 (PMC12167381; doi:10.1038/s41746-025-01692-1)
Supplement: Supplementary file 1 — Supplementary Information [file 41746_2025_1692_MOESM1_ESM.pdf]

A SCOPING REVIEW OF SELF-SUPERVISED REPRESENTATION LEARNING FOR CLINICAL DECISION MAKING USING EHR CATEGORICAL DATA

Supplementary Table 1. Data item used in full-text charting

Category	Item	Description
Article information	Title of article	Title of article
	Publication year	Year of publication
	Publication journal	Journal in which the studies were published
	Number of citations	Number of citations by July 2024
Authorship information	Research team composition	The composition of authors (data scientist and medical expert)
	Authors	First author name
	International collaboration	If the authors originated from reasearch instituts of different countries
	Medical expert contribution	The involvement of medical experts in the study
Clinical data information	Data availability	If the dataset used in the study accessible by public
	Data source	Name of dataset or the clinical institutes where the data were collected
	Data deidentification	If the clinical data were deidentified for privacy protection
	Patient cohort selection criteria	The inclusion / exclusion criteria for patient cohort selection
	Patient type for SSRL training	The characteristics or medical conditions of patients used for pretraining the SSRL model.
	Number of patients for SSRL training	The total number of patients included in the pretraining phase of the SSRL model.
	External medical knowledge (if any)	Additional medical knowledge or external datasets used to improve the model training process.
SSRL training	Used data type for model training	Types of data used for training, including categorical data with coding systems and numerical data, if applicable.
	Data preprocessing techniques	Methods applied to prepare data for model training, including data augmentation, transformation, normalization, and feature engineering.
	SSRL temporality modeling	Indicates whether the model accounts for the temporal aspects of the data and specifies the level of granularity at which time is modeled.
	SSRL model type	The specific type or architecture of SSRL model used in the study
Downstream task	Downstream task model type	Type of model used for downstream tasks
	Downstream clinical task type	The category of clinical tasks performed by the model, such as clustering, classification, or regression.

	Downstream clinical task domain	The specific medical or clinical field in which the downstream task is applied
	Downstream clinical task	The clinical task
	Evaluation metrics	The specific clinical application or objective of the model
Computational requirement	Computational resources requirement	The hardware and training time needed for model training and deployment
Transparency	Interpretability	Interpretability techniques used in the study
Transferability of model	Prove of model transferability	Empirical validation showing that the model maintains its performance when applied to different datasets or clinical settings.
	External dataset for transferability	Indicates whether external datasets were used to assess the model's transferability and generalization capabilities.

Supplementary Table 2. Search Strategy: Database-specific search strategy. The search queries and the number of used in the corresponding databases.

Database	Query	Result
PubMed	("deep learning" [tiab] OR "neural network"[tiab] OR "machine learning"[tiab]) AND ("unsupervised"[tiab] OR "self-supervised"[tiab] OR "pretrain*"[tiab] OR "pre-train"[tiab] OR "BERT"[tiab]) AND ("electronic health record?"[tiab] OR "ehr" OR "electronic medical record?"[tiab] OR "emr"[tiab] OR "Electronic Health Records"[Mesh] OR "health care data"[tiab] OR "patient longitudinal"[tiab]) AND 2019/01/01:2024/12/31[pdat]	306
Web of science	(TI=(("deep learning" OR "neural network" OR "machine learning") AND ("unsupervised" OR "self-supervised" OR "pretrain*" OR "pre-train*" OR "BERT") AND ("electronic health record?" OR "ehr" OR "electronic medical record?" OR "emr" OR "Electronic Health Records" OR "health care data" OR "patient longitudinal" OR "patient trajectory"))) OR AB=(("deep learning" OR "neural network" OR "machine learning") AND ("unsupervised" OR "self-supervised" OR "pretrain*" OR "pre-train*" OR "BERT") AND ("electronic health record?" OR "ehr" OR "electronic medical record?" OR "emr" OR "Electronic Health Records" OR "health care data" OR "patient longitudinal" OR "patient trajectory"))	180
ACM	Title:(("deep learning" OR "neural network" OR "machine learning") AND ("unsupervised" OR "self-supervised" OR "pretrain*" OR "pre-train*" OR "BERT") AND ("electronic	45

	health record?" OR "ehr" OR "electronic medical record?" OR "emr" OR "Electronic Health Records" OR "health care data" OR "patient longitudinal"))) OR Abstract:(("deep learning" OR "neural network" OR "machine learning") AND ("unsupervised" OR "self-supervised" OR "pretrain*" OR "pre-train*" OR "BERT") AND ("electronic health record?" OR "ehr" OR "electronic medical record?" OR "emr" OR "Electronic Health Records" OR "health care data" OR "patient longitudinal")))	
Embase and MEDLINE	('deep learning':ti,ab,kw OR 'neural network':ti,ab,kw OR 'machine learning':ti,ab,kw) AND ('unsupervised':ti,ab,kw OR 'self-supervised':ti,ab,kw OR 'pretrain*':ti,ab,kw OR 'pre-train*':ti,ab,kw OR 'bert':ti,ab,kw) AND ('electronic health record*':ti,ab,kw OR 'ehr' OR 'electronic medical record*':ti,ab,kw OR 'emr':ti,ab,kw OR 'electronic health record'/exp OR 'health care data':ti,ab,kw OR 'patient longitudinal':ti,ab,kw OR 'patient trajectory':ti,ab,kw) AND [2019-2024]/py	347

Supplementary Table 3. Preferred Reporting Items for Systematic reviews and Meta-Analyses extension for Scoping Reviews (PRISMA-ScR) Checklist

SECTION	ITEM	PRISMA-ScR CHECKLIST ITEM	Reported in Section
TITLE			
Title	1	Identify the report as a scoping review.	Title page
ABSTRACT			
Structured summary	2	Provide a structured summary that includes (as applicable): background, objectives, eligibility criteria, sources of evidence, charting methods, results, and conclusions that relate to the review questions and objectives.	Abstract
INTRODUCTION			
Rationale	3	Describe the rationale for the review in the context of what is already known. Explain why the review questions/objectives lend themselves to a scoping review approach.	Introduction
Objectives	4	Provide an explicit statement of the questions and objectives being addressed with reference to their key elements (e.g., population or participants, concepts,	Introduction

		and context) or other relevant key elements used to conceptualize the review questions and/or objectives.	
METHODS			
Protocol and registration	5	Indicate whether a review protocol exists; state if and where it can be accessed (e.g., a Web address); and if available, provide registration information, including the registration number.	N/A
Eligibility criteria	6	Specify characteristics of the sources of evidence used as eligibility criteria (e.g., years considered, language, and publication status), and provide a rationale.	Methods
Information sources*	7	Describe all information sources in the search (e.g., databases with dates of coverage and contact with authors to identify additional sources), as well as the date the most recent search was executed.	Methods
Search	8	Present the full electronic search strategy for at least 1 database, including any limits used, such that it could be repeated.	Supplementary Table 2
Selection of sources of evidence	9	State the process for selecting sources of evidence (i.e., screening and eligibility) included in the scoping review.	Methods
Data charting process	10	Describe the methods of charting data from the included sources of evidence (e.g., calibrated forms or forms that have been tested by the team before their use, and whether data charting was done independently or in duplicate) and any processes for obtaining and confirming data from investigators.	Methods
Data items	11	List and define all variables for which data were sought and any assumptions and simplifications made.	Supplementary Table 1
Critical appraisal of individual sources of evidence	12	If done, provide a rationale for conducting a critical appraisal of included sources of evidence; describe the methods used and how this information was used in any data synthesis (if appropriate).	N/A
Synthesis of results	13	Describe the methods of handling and summarizing the data that were charted.	Methods
RESULTS			
Selection of sources of evidence	14	Give numbers of sources of evidence screened, assessed for eligibility, and included in the review, with reasons for exclusions at each stage, ideally using a flow diagram.	Methods

Characteristics of sources of evidence	15	For each source of evidence, present characteristics for which data were charted and provide the citations.	Result
Critical appraisal within sources of evidence	16	If done, present data on critical appraisal of included sources of evidence (see item 12).	N/A
Results of individual sources of evidence	17	For each included source of evidence, present the relevant data that were charted that relate to the review questions and objectives.	Result and Supplementary Data 1-8
Synthesis of results	18	Summarize and/or present the charting results as they relate to the review questions and objectives.	Result
DISCUSSION			
Summary of evidence	19	Summarize the main results (including an overview of concepts, themes, and types of evidence available), link to the review questions and objectives, and consider the relevance to key groups.	Discussion
Limitations	20	Discuss the limitations of the scoping review process.	Discussion
Conclusions	21	Provide a general interpretation of the results with respect to the review questions and objectives, as well as potential implications and/or next steps.	Discussion
FUNDING			
Funding	22	Describe sources of funding for the included sources of evidence, as well as sources of funding for the scoping review. Describe the role of the funders of the scoping review.	Acknowledgements

Supplementary Data

Supplementary Data 1 - This table illustrates the relationship between the clinical event being predicted or visualized and their corresponding clinical domains.

Supplementary Data 2 - This table illustrates the basic information of included studies: publication date, published journal, authors and research team information and dataset used in the study.

Supplementary Data 3 - This table illustrates the data modalities used in the studies included. Various data types can be used in the studies: categorical data, numerical data, and others such as clinical text and images.

Supplementary Data 4 - This table illustrates the data preprocessing of each study, the methods used for feature selection and the number of concepts / tokens in the original data versus after the data preprocessing.

Supplementary Data 5 - This table illustrates the SSRL model types used in the included studies, and the inferred temporality.

Supplementary Data 6 - This table illustrates the information of the patient cohorts used for SSRL model training, criteria of inclusion/exclusion for patient selection and the data privacy for included studies.

Supplementary Data 7 - This table illustrates the detailed downstream tasks and the interpretability of the included studies.

Supplementary Data 8 - This table illustrates the used computational resources and transferability of included studies.
